# Hop2 interacts with the transcription factor CEBPα and suppresses adipocyte differentiation

**DOI:** 10.1016/j.jbc.2021.101264

**Published:** 2021-09-30

**Authors:** Tonghui Lin, Yang Zhang, Tingting Zhang, Rita A. Steckler, Xiangli Yang

**Affiliations:** Department of Pediatrics, Pediatric Research Center, University of Texas Health Science Center at Houston, McGovern Medical School, Houston, Texas, USA

**Keywords:** differentiation, adipocytes, transcription factor, CEBPα, Hop2, Ab, antibody, aMSC, adipocyte-derived mesenchymal stem cell, *An*, adiponectin, *aP2*, adipocyte protein 2, ATF4, activating transcription factor 4, *C3*, complement 3, *CD36*, cluster of differentiation 36, cDNA, complementary DNA, CEBP, CCAAT enhancer binding protein, ChIP, chromatin immunoprecipitation, co-IP, coimmunoprecipitation, *Dgat*, diglyceride acyltransferase, *Glut4*, glucose transporter 4, GST, glutathione-*S*-transferase, HA, hemagglutinin, HA-Hop2, HA-tagged Hop2, het, heterozygous, Hop2, homologous pairing protein 2, *Lep*, leptin, NE, nuclear extract, Neo, neomycin-resistant gene, ON, overnight, ORO, Oil Red O, *Pepck*, phosphoenolpyruvate carboxykinase, PPARγ, peroxisome proliferator–activated receptor γ, qRT–PCR, quantitative RT–PCR, TF, transcription factor, VAT, visceral adipose tissue

## Abstract

CCAAT enhancer binding protein (CEBP) transcription factors (TFs) are known to promote adipocyte differentiation; however, suppressors of CEBP TFs have not been reported thus far. Here, we find that homologous chromosome pairing protein 2 (Hop2) functions as an inhibitor for the TF CEBPα. We found that *Hop2* mRNA is highly and specifically expressed in adipose tissue, and that ectopic Hop2 expression suppresses reporter activity induced by CEBP as revealed by DNA transfection. Recombinant and ectopically expressed Hop2 was shown to interact with CEBPα in pull-down and coimmunoprecipitation assays, and interaction between endogenous Hop2 and CEBPα was observed in the nuclei of 3T3 preadipocytes and adipocytes by immunofluorescence and coimmunoprecipitation of nuclear extracts. In addition, Hop2 stable overexpression in 3T3 preadipocytes inhibited adipocyte differentiation and adipocyte marker gene expression. These *in vitro* data suggest that Hop2 inhibits adipogenesis by suppressing CEBP-mediated transactivation. Consistent with a negative role for Hop2 in adipogenesis, ablation of *Hop2* (*Hop2*^−/−^) in mice led to increased body weight, adipose volume, adipocyte size, and adipogenic marker gene expression. Adipogenic differentiation of isolated adipose-derived mesenchymal stem cells showed a greater number of lipid droplet–containing colonies formed in *Hop2*^−/−^ adipose-derived mesenchymal stem cell cultures than in wt controls, which is associated with the increased expression of adipogenic marker genes. Finally, chromatin immunoprecipitation revealed a higher binding activity of endogenous CEBPα to peroxisome proliferator–activated receptor γ, a master adipogenic TF, and a known CEBPα target gene. Therefore, our study identifies for the first time that Hop2 is an intrinsic suppressor of CEBPα and thus adipogenesis in adipocytes.

Adipose tissues play a significant role in the maintenance of metabolic homeostasis. It stores energy in the form of fat, offers insulation and protection to soft vital organs, and produces more than 600 secretory molecules, collectively named adipokines, to regulate food intake, energy expenditure, insulin sensitivity, fat distribution, inflammation, and blood pressure (1, 2). However, excessive fat accumulation in obese individuals is a serious pandemic currently affecting 42% of adult Americans (3). Obesity increases the risk for many diseases, including cardiovascular disease, stroke, type 2 diabetes, physical disability (4, 5), and cancer (6). The cellular basis of obesity involves an increased number and size of adipocytes, resulting from proliferation and differentiation of adipocyte progenitor cells or preadipocytes (7, 8). Adipocytes contribute more than 90% of the adipose tissue volume in the human body and other vertebral animals (9). Understanding the molecular and cellular basis of adipocyte proliferation and differentiation is critical to improving the management of obesity and associated diseases.

The molecular regulation of adipocyte differentiation has been extensively investigated. A transcriptional cascade, which was identified 3 decades ago by *in vitro* studies of the cultured preadipocyte 3T3-L1 cell line (10, 11), plays a central role in the regulation of this process. Upon the stimulation of an adipogenic “cocktail” containing insulin and glucocorticoids, two basic (b) leucine zipper (Zip) containing transcription factors (TFs), the CCAAT enhancer binding protein (CEBP) β and CEBPδ, are expressed. The two CEBP proteins form a heterodimer to bind promoters of the peroxisome proliferator–activated receptor γ (PPARγ), a member of the nuclear receptor superfamily of ligand-activated TFs, and CEBPα. The PPARγ and CEBPα can activate themselves and each other to form a feed-forward loop that maintains a high level of PPARγ expression in terminally differentiated adipocytes (12). The PPARγ and CEBPα have been shown by RNA sequencing and chromatin immunoprecipitation (ChIP) followed by deep sequencing (ChIP-Seq) to synergistically activate many key metabolic adipocyte marker genes, including adipocyte protein 2 (*aP2*), glucose transporter 4 (*Glut4*), phosphoenolpyruvate carboxykinase (*Pepck*), leptin (*Lep*), and adiponectin (*An*) involved in fatty acid transport, triglyceride synthesis, and insulin sensitivity (13). These *in vitro* findings are supported by genetic data that *CEBPβ*^−/−^ mice display reduced body fat mass and resist to diet-induced obesity (14, 15), and CEBPβ/δ double mutant mice have a greater reduction in body adiposity, accompanied with decreased expression of CEBPα and PPARγ transcripts (16, 17). Similarly, *CEBPα*^−/−^ or *PPARγ*^−/−^ mice have no white adipose, and their adipocytes fail to accumulate lipid and express adipocyte marker genes, whereas cell lineage commitment is not affected (12, 17, 18, 19). Thus, both *in vitro* and *in vivo* studies demonstrated this cascade of TFs in which the CEBPβ and CEBPδ activate PPARγ and CEBPα to promote adipocytes to acquire and maintain terminally differentiated phenotypes.

In addition to the CEBPs, activating transcription factor 4 (ATF4), a member of the ATF/CREB subfamily of the bZip superfamily subfamilies (20), has also been shown to promote adipogenesis by dimerizing with CEBPβ in both human mesenchymal stem cell and 3T3-L1 preadipocyte cultures (21, 22). Supporting these *in vitro* findings, *Atf4*^−/−^ mice are lean and resist diet-induced obesity (23, 24, 25, 26, 27). Using the bZip motif as a bait, we recently identified that homologous pairing protein 2 (Hop2) also binds to ATF4 (28). Hop2 was originally identified in yeast as a meiosis-specific protein required for interchromosomal interaction/paring during meiosis (29). A human homolog of Hop2 has been identified by its suppression of the HIV replication suppressor TBP1 (30, 31). Somatic mutations in *Hop2* are prevalent in sporadic breast, ovarian, and fallopian tube cancer (32, 33). In mammals, the mRNA of *Hop2* is robustly expressed in the testis and genetic ablation of *Hop2* in mice, in which the first three exons of the *Hop2* gene were replaced with the neomycin-resistant gene (Neo), leads to infertility because of a failure in the formation of haploid gametes (34). Previous cell culture studies found a nonmeiotic role that Hop2 plays by acting as a tissue-specific coactivator of nuclear receptors, which is consistent with the observation that the expression of *Hop2* mRNA was broader than originally reported (28).

Our previous work demonstrated that in osteoblasts, the binding of Hop2 to ATF4 stimulates ATF4-mediated transactivation and promotes osteoblast differentiation *in vitro* and *in vivo*. Genetic interaction between ATF4 and Hop2 was best demonstrated by osteopenia (low bone mass) in the *Atf4*^+/−^:*Hop2*^+/−^ double heterozygous (het) mice, which is identical to a low bone mass in *Hop2*^−/−^ mice and milder than osteoporosis in *Atf4*^−/−^ mice (23, 28). Interestingly, *Hop2*^−/−^ mice display an increased body weight and adipose tissue mass under normal chow, and this abnormal adipose phenotype is absent in the *Atf4*^+/−^:*Hop2*^+/−^ double het mice. This important observation led us to hypothesize that *Hop2* in adipocytes interacts with one or more of the three adipocyte bZip TFs to regulate adipogenesis. This hypothesis is further supported by our previous finding that ATF4 is an extremely labile protein that is undetectable in adipose tissue (35, 36, 37).

In this study, we identified another Hop2-interacting protein, CEBPα, in adipocytes. We provide data demonstrating that the binding of Hop2 to CEBPα suppresses the DNA binding of CEBPα to its target genes. *In vivo*, ablation of *Hop2* in mice leads to increased adiposity compared with wt or het (+/−) littermates, whereas, in cells, forced expression of Hop2 in preadipocytes suppresses adipocyte differentiation. Consistent with an inhibitory role, the Hop2 protein level is high in preadipocytes but low in differentiated adipocytes. Thus, our study for the first time reveals a novel function of Hop2 in adipose tissues.

## Results

### Hop2 is highly and specifically expressed in adipocytes

**Figure 1:**
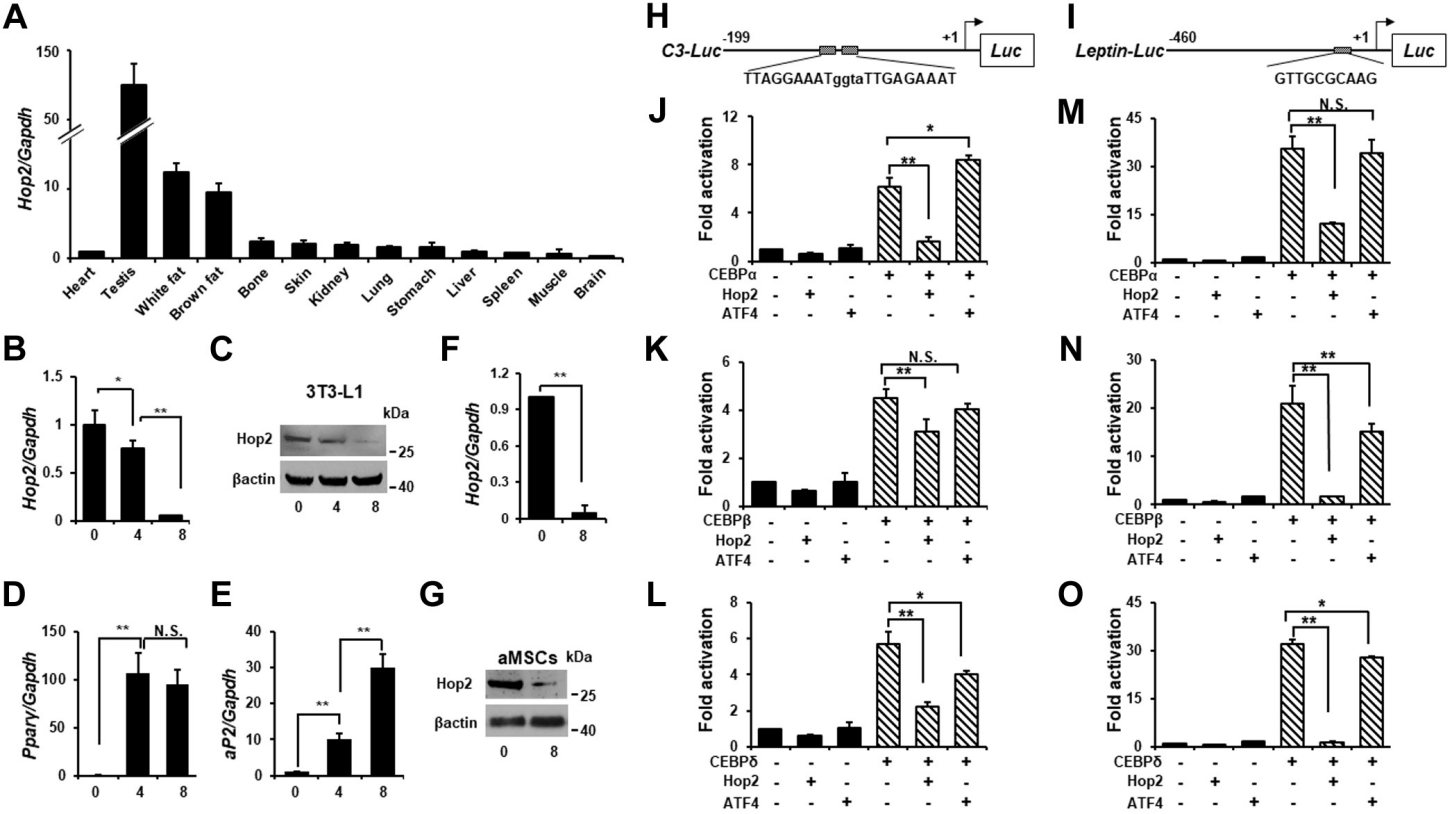
**Hop2 is expressed in adipocytes and inhibits CEBPα-mediated transcription.***A*, qPCR analysis of *Hop2* expression in indicated tissues and organs from 2-month-old male mice. Data are shown as mean and standard deviation of three samples. Primers used for all qRT–PCR analyses are listed in Table 1. n = 3 unless indicated. *B*, qPCR analysis for *Hop2* expression in differentiating 3T3-L1 adipocytes. The *numbers* indicate cell cultured days following adipogenic induction at day 0 (defined as 2 days after cells reaching confluency). ∗*p* < 0.05 and ∗∗*p* < 0.01 for all panels. *C*, Western blot analysis of nuclear extracts from differentiating 3T3-L1 adipocytes. Culture condition is detailed in *B*, and β-actin is served as a loading control. *D* and *E*, qPCR analysis for *Pparγ* and *aP2* expression in differentiating 3T3-L1 adipocytes. Culture condition is detailed in *B* and *C*. *F*, qPCR analysis for *Hop2* expression in primary adipocyte-derived mesenchymal stem cells (aMSCs) during adipogenic differentiation. Confluent aMSCs (day 0) of passage 1 were induced to differentiate with adipogenic cocktail for 8 days. *G*, Western blot analysis of nuclear extracts from differentiating aMSCs as described in *F*. *H*, schematic presentation of *C3-Luc* reporters used in DNA transfection assays shown in *J*–*L*. Two AT-rich consensus sequences in a tandem repeat in *C3-Luc* to all the CEBP transcription factors (TFs) are indicated with *uppercase letters*. *I*, schematic presentation of *Leptin-Luc* reporters used in DNA transfection assays shown in *M*–*O*. One G-rich consensus sequence in *Leptin-Luc* common to all the CEBP TFs is indicated with *uppercase letters*. *J*–*O*, DNA cotransfection assays in COS1 cells with reporter constructs (*H* and *I*) and expression plasmids for Hop2 and indicated bZip TFs. ATF4 as a subfamily member of the ATF/Creb bZip subfamily serves as a control for the members of the tested adipocyte CEBP bZip subfamily. CEBPα, CCAAT enhancer binding protein alpha; qPCR, quantitative PCR.

With the C-terminal bZip motif of ATF4 on a yeast hybrid screening of mouse complementary DNA (cDNA) library, we previously identified an atypical Zip-containing protein Hop2 that binds and stimulates ATF4-dependent transcription and osteoblast differentiation (28). Intriguingly, our data revealed that this well-characterized meiotic-specific protein is also highly and specifically expressed in adipose tissue among 13 somatic tissues examined (28). Quantitative RT–PCR (qRT–PCR) of total RNA isolated from 2-month-old male mouse tissues revealed that *Hop2* mRNA level is about 10 and 12 times higher in brown and white fat adiposes than in skin, bone, lung, and kidney, but approximately ten times lower than in testis (Fig. 1*A*). The endogenous expression of Hop2 in preadipocytes and adipocytes was then validated by qRT–PCR and Western blot with validated antibody (Fig. S1) analyses of differentiating primary adipocyte-derived mesenchymal stem cells (aMSCs) and 3T3-L1 preadipocytes, and interestingly, *Hop2* mRNA and protein levels are higher in undifferentiated 3T3-L1 preadipocytes than in fully differentiated adipocytes (Fig. 1, *B*–*G*). These results suggest an intrinsic role that Hop2 plays in the regulation of adipocyte differentiation and function.

### Hop2 specifically suppresses CEBP-mediated transactivation potential

To begin to address the functional relevance of the expression of Hop2 in adipocyte differentiation, we tested whether Hop2 influences the CEBP-dependent transactivation activity *in vitro*. The three adipocyte CEBP isoforms, CEBPα, CEBPβ, and CEBPδ, share over 90% homology in their DNA-binding domains and recognize and activate the same consensus sequences (38). Two reporter constructs, *C3-Luc* and *Leptin-Luc*, were used in our DNA cotransfection assays. The complement 3 (*C3*) contains a promoter fragment covering −1 to −199 bp of the human *C3* with two direct repeats of a CEBP consensus binding sequence at −110 (TTGAGAAAT) and −123 (TTAGGAAAT) (39, 40) (Fig. 1*H*), respectively, whereas the *Leptin-Luc* reporter construct contains a 456-bp mouse *Leptin* proximal promoter (Fig. 1*I*) with a perfect CEBPα binding palindrome (TTGCGCAA) (41). Reporter assays showed that cotransfection of CEBPα, CEBPβ, and CEBPδ expression plasmids, individually, with the *C3-Luc* increases the reporter's activity by 6.1-, 4.5-, and 5.7-fold, respectively. Hop2 cotransfection brought down the CEBP-induced reporter's activity to 1.6-, 3.1-, and 2.2-fold, corresponding to a 74%, 31%, and 61% suppression on CEBPα-, CEBPβ-, and CEBPδ-dependent activation, respectively (Fig. 1, *J*–*L*). This inhibitory effect is specific for Hop2 because cotransfection of ATF4, a bZip TF reported dimerizing with CEBPβ (21, 22) and Hop2 (28), had no significant suppression on the CEBPα- and CEBPβ-dependent reporter's activity, except for a 30% inhibition on CEBPδ-induced transactivation. The Hop2-specific suppression on the CEBP-mediated transactivation was also observed on the *Leptin-Luc* reporter, in which Hop2 reduces CEBP-induced reporter's activity from 35.7- to 12.2-fold, 21.0- to 1.7-fold, and 31.9- to 1.4-fold representing a 66%, 92%, and 97% inhibition for CEBPα, CEBPβ, and CEBPδ, respectively (Fig. 1, *M*–*O*). Again, ATF4 cotransfection did not cause a significant change in the CEBP-dependent activation. These data indicated that Hop2 conveys a strong and specific inhibition of the transactivation potential of all three CEBP isoforms.

### Hop2 interacts with CEBPα in differentiating adipocytes

**Figure 2:**
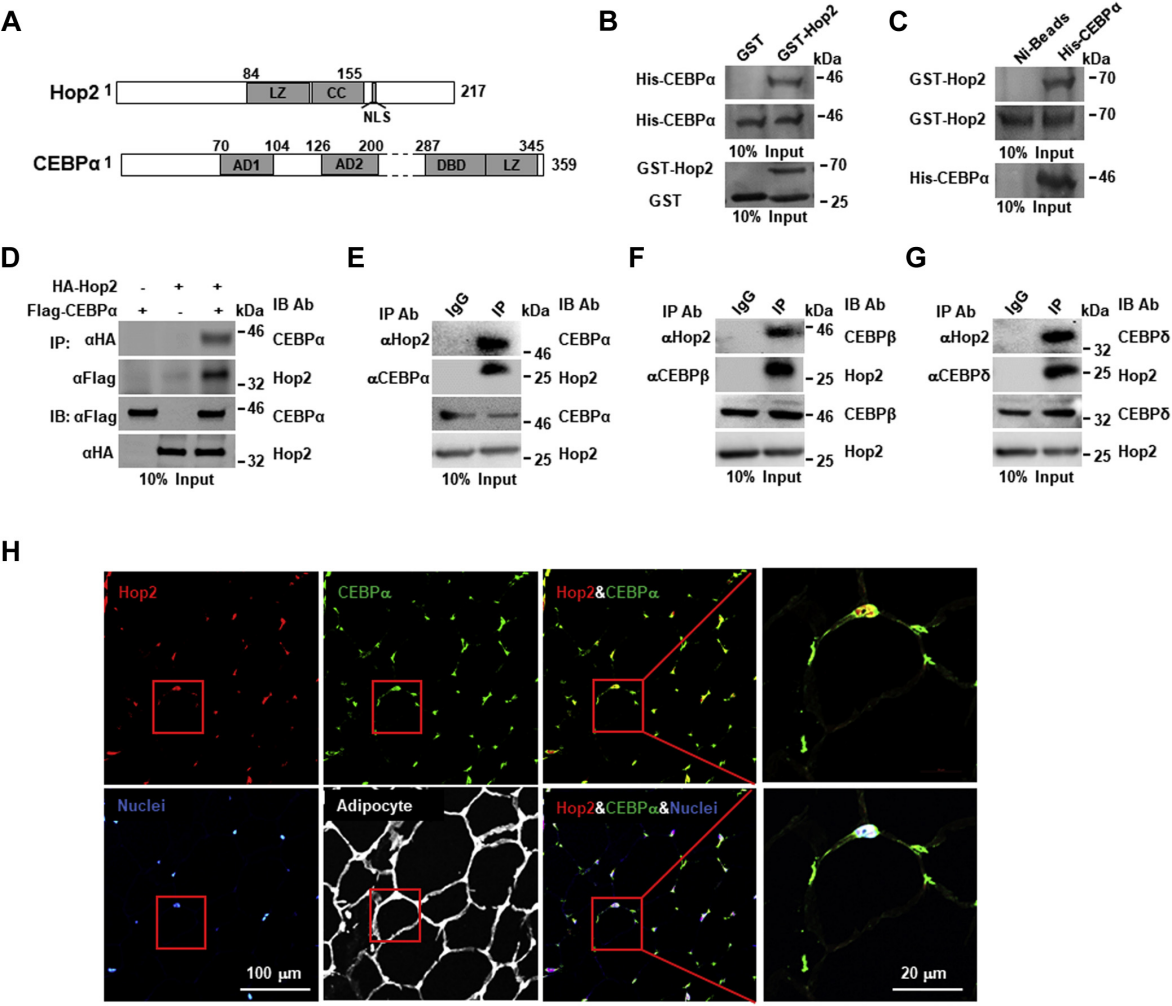
**Hop2 interacts with adipocyte CEBP.***A*, schematic illustration of CEBPα as a representative of adipocyte CEBPs (α, β, and δ) and Hop2 primary structure. Amino acid residues are indicated by *numeric letters*. *B*, pull-down assays for a direct interaction between bacterially expressed GST-Hop2 and His-CEBPα fusions. Purified His-CEBPα was incubated with GST-Hop2 or GST-bound glutathione agarose. The proteins retained were resolved with SDS-gel and visualized by Coomasie blue staining. *C*, pull-down assays. Ni-affinity beads were incubated with a mixture of His-CEBPα and GST-Hop2 or GST-Hop2 alone (negative control). The bound proteins were resolved with SDS-gel and visualized by Coomasie blue staining. *D*, coimmunoprecipitation (co-IP) assays for the interaction between HA-tagged Hop2 and Flag-tagged CEBPα. Nuclear extracts (NEs) from COS1 cells transfected with HA-Hop2 and/or with anti-HA or anti-Flag antibodies (Abs). Western blot analysis of precipitates for Flag and HA (*top two panels*) and NE input (10%) for each co-IP (*bottom panel*). *E*, co-IP and Western blot analysis of NEs from wt mouse visceral adipose tissue (VAT) for endogenous interaction (*top two panels*) and expression of Hop2 and CEBPα (*bottom two panels*). Abs used for IP and IB are indicated. *F*, co-IP and Western blot analysis of NEs from wt mouse VAT for endogenous interaction (*top two panels*) and expression of Hop2 and CEBPβ (*bottom two panels*). Abs used for IP and IB are indicated. *G*, co-IP and Western blot analysis of NEs from wt mouse VAT for endogenous interaction (*top two panels*) and expression of Hop2 and CEBPδ (*bottom two panels*). Abs used for IP and IB are indicated. *H*, immunofluorescence for colocalization of Hop2 and CEBPα in nuclei of adipocytes. Sections of paraffin-embedded mouse adipose tissues were stained with Abs indicated on the *top left* of each image. Nuclei are revealed with Hoechst staining. AD, activation domain; CC, coiled-coil motif; CEBP, CCAAT enhancer binding protein; DBD, DNA-binding domain; GST, glutathione-*S*-transferase; LZ, leucine zipper domain; NLS, nuclear location signal (EWRKRKR).

Leucine zipper domain or Zip is a common structural motif mediating protein–protein interactions (42), and our previous studies established that the Zip domain of Hop2 is necessary and sufficient to mediate its interaction with the bZip domains of ATF4 (28). These findings led us to hypothesize that Hop2 suppresses the CEBP-dependent transactivation by interacting with these bZip TFs in adipocytes. Given the fact that genetic deletion of CEBPα alone was sufficient to block adipocyte differentiation *in vivo* (17), we focused our next set of experiments on this founding member of the CEBP subfamily (43) (Fig. 2*A*). To detect a direct binding between Hop2 and CEBPα, we purified recombinant glutathione-*S*-transferase (GST)-Hop2 and His-CEBPα fusion proteins expressed in bacteria. Pull-down assays demonstrated that GST-Hop2, but not GST, was able to pull down His-CEBPα protein, and conversely, a Ni-charged affinity chromatography retained GST-Hop2 only in the presence of His-CEBPα (Fig. 2, *B* and *C*). These results thus show that Hop2 and CEBPα interact directly *in vitro*. To determine whether Hop2 interacts with CEBPα in cells, we transfected COS1 cells transiently with expressing plasmids encoding hemagglutinin (HA)-tagged Hop2 (HA-Hop2) and Flag-CEBPα singly or together. Nuclear extracts (NEs) of COS1 cells ectopically expressing HA-Hop2 and Flag-CEBPα were subjected to coimmunoprecipitation (co-IP). As expected, an anti-HA antibody (Ab) precipitated HA-Hop2 and an anti-Flag Ab precipitated Flag-CEBPα from NEs of COS1 cells expressing either HA-Hop2 or Flag-CEBPα alone (positive control; Fig. 2*D*). Moreover, the anti-HA cross precipitated Flag-CEBPα, whereas the anti-Flag Ab brought down HA-Hop2 from the NEs of COS1 cells coexpressing both HA-Hop2 and Flag-CEBPα, but not expressing each of these proteins singly (negative controls). Furthermore, immunocytochemistry of COS1 cells transiently overexpressing both HA-Hop2 and Flag-CEBPα and of 3T3-L1 preadipocytes transiently overexpressing HA-Hop2 detected double-positive signals (*orange*) with anti-HA (*green*) and anti-Flag (*red*) or anti-CEBPα Abs in the nuclei of these cells (Fig. S2, *A* and *B*), confirming a colocalization of Hop2 and CEBPα when overexpressed.

To further examine whether endogenous Hop2 interacts with adipocyte CEBP bZip TFs under physiological conditions, we isolated NEs from visceral adipose tissues (VATs) and performed co-IP with Abs against Hop2 and CEBP bZips. Anti-Hop2 Abs, but not anti-IgG Abs (negative control), precipitated all three isoforms of the CEBPα, CEBP-β, and CEBP-δ; and reciprocally, anti-CEBPα, anti-CEBPβ, and anti-CEBPδ Abs, but not anti-IgG Abs, precipitated Hop2 (Fig. 2, *E*–*G*). In addition, immunofluorescence of mouse VAT verified that endogenous Hop2 (*red*) and CEBPα (*green*) are colocalized in the nuclei (*blue*) of adipocytes (Fig. 2*H*), supporting that Hop2 plays an intrinsic physiological role in adipocytes.

To further explore at which stage(s) of adipocyte differentiation that the interaction between Hop2 and CEBPs takes place, we analyzed the temporal and dynamic expression pattern of the three CEBP bZip TFs. Western blot found that the protein expression levels of CEBPβ and CEBP-δ in 3T3-L1 cells are low in undifferentiated cultures (day 0), increased in 2-day cultures, and then reduced to undetectable in 4-day cultures upon adipocyte induction (Fig. S2*C*). On the other hand, CEBPα protein expression is undetectable in day 0, induced in day 4, and then disappeared at day 8 cultures following adipogenic induction (Fig. S2*D*). These expression patterns of the adipocyte bZip TFs are consistent with previous reports (10) and support the well-known role of the cascade of bZip TFs in promoting adipocyte differentiation. Based on these expression profiles, we carried out co-IP on NEs of differentiated 3T3-L1 cultures and observed that anti-CEBPβ and anti-CEBPδ Abs precipitated endogenous Hop2 in the NEs of day 2 cultures (Fig. S2, *F* and *G*), whereas anti-CEBPα Abs precipitated Hop2 in NEs of day 4 cultures. Reciprocally, anti-Hop2 Abs precipitated CEBPβ and CEBPδ in the NEs of day 2 cultures, whereas CEBPα in the NEs of day 4 cultures (Fig. S2*E*). Collectively, our exhaustive dataset strongly supports the hypothesis that Hop2 inhibits CEBP-mediated transactivation of target genes by direct interaction.

### Hop2 gain-of-function delays adipocyte differentiation

**Figure 3:**
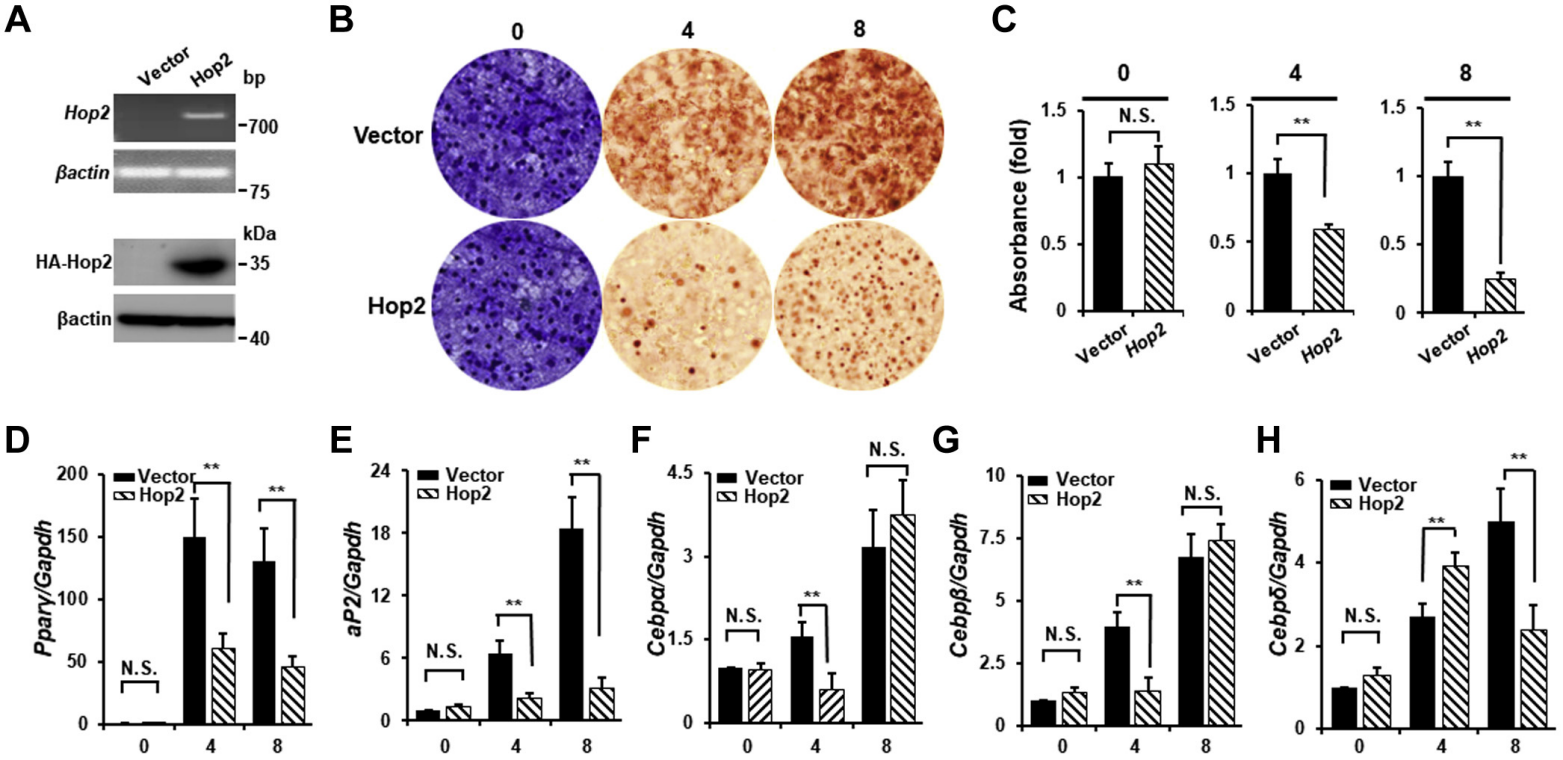
**Stable Hop2 overexpression in preadipocytes inhibits adipocyte differentiation.***A*, RT–PCR of RNA (*upper two panels*) and Western blot of nuclear extracts (*lower two panels*) from 3T3-L1 preadipocytes stably expressing *Hop2*. *B*, adipocyte differentiation assays. Crystal violet staining (*left panels*) for equal seeding density of stable Hop2-expressing 3T3-L1 cells and day 0 was defined as described in *C*. ORO staining (*middle* and *right panels*) for lipid-containing differentiated colonies at indicated days following adipogenic induction. *C*, quantification of eluted dye from stained cells in *B*. Absorption of the eluate was measured photometrically at 570 nm for crystal violate and at 510 nm for ORO. *D*–*H*, quantitative RT–PCR analysis for adipocyte marker genes in differentiating 3T3-L1 cells stably expressing Hop2. ORO, Oil Red O.

To assess whether Hop2 is sufficient to impact adipocyte differentiation, we created stable lines of 3T3-L1 preadipocytes overexpressing either HA-Hop2 or empty vector as a control. The HA-Hop2 expression was validated by RT–PCR for the mRNA and by Western blot analysis using Abs against HA tag for the HA-Hop2 protein (Fig. 3*A*). Adipocyte differentiation of these cell lines was then assayed on three time points at day 0 (defined by 2 days after cells reached confluency), day 4, and day 8 following adipogenic cocktail addition by Oil Red O (ORO) staining and qRT–PCR of adipocyte marker genes. As shown in Figure 3*B*, the number and size of colonies containing lipid droplets with ORO positivity (ORO+) are higher and larger in the control cells expressing empty vector (control) than in cells expressing HA-Hop2 in both day 4 and day 8 cultures (Fig. 3*B*). Spectrophotometry quantification measuring absorbance at 570 nm for ORO released from cultures revealed a 40% and 75% reduction by HA-Hop2 overexpression upon a differentiation of 4 and 8 days, respectively (Fig. 3*C*). Accordingly, qRT–PCR found that the expression level of two late adipocyte marker genes is decreased by 60% and 65% for PPARγ as well as 67% and 93% for *aP2* in cultures of HA-Hop2 expressing 3T3-L1 cells on days 4 and 8, respectively. A reduction in the expression level of adipocyte bZip genes was only observed one time point either in day 4 or day 8 cultures of the HA-Hop2 expressing cells with a 65% decrease for CEBPα, 61% for CEBPβ in day 4, and 54% for CEBPδ in day 8 cultures, respectively (Fig. 3, *D*–*H*). In summary, Hop2 gain-of-function studies in 3T3-L1 preadipocytes are sufficient to suppress the progression of adipocyte differentiation.

### Hop2 ablation in mice leads to increased adipose volume

**Figure 4:**
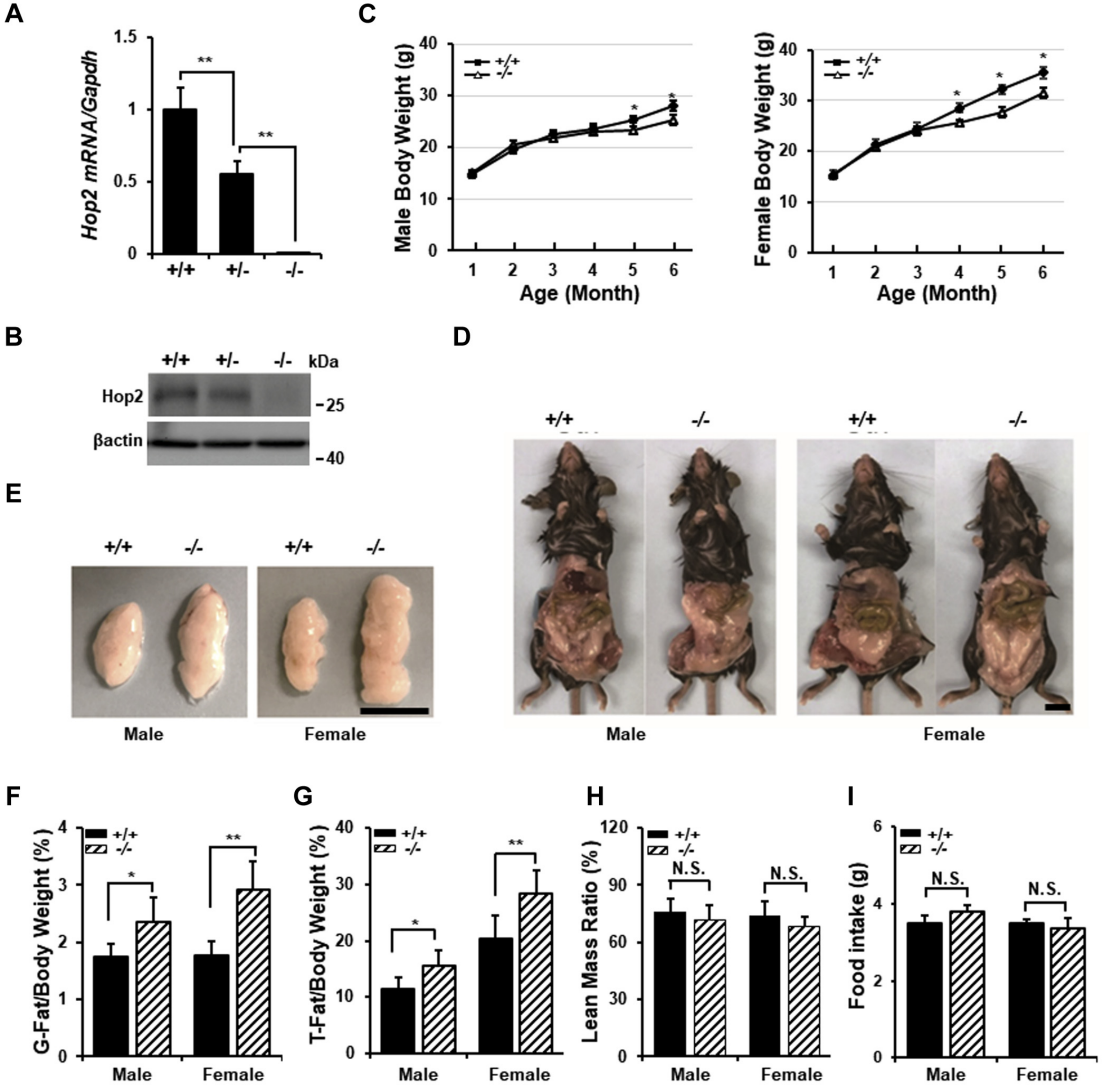
***Hop2***^**−/−**^**mice exhibit increased fat mass.***A*, quantitative PCR analysis for *Hop2* mRNA of RNA isolated from visceral adipose of 2-month-old male mice. Data are shown as mean ± standard deviation of three samples per genotype. *B*, Western blot analysis of nuclear extracts from visceral adipose of 2-month-old male mice. Data shown are representatives of three mice per genotype. *C*, growth chart for male (*left*) and female (*right*) mice of indicated genotype. Data are shown as mean ± standard deviation of four mice per genotype. *Asterisks* indicate significant differences in all panels (∗*p* < 0.05; ∗∗*p* < 0.01). *D*–*F*, representative image of gonadal adipose from 6-month-old mice of indicated sex and genotype (*D* and *E*). The ratio of gonadal adipose (*F*) over total body weight is shown as the mean ± standard deviation of 7 to 9 mice per group. The *black bars* equal 1 cm. *G* and *H*, echo-MRI scans quantification of adipose and lean mass from 6-month-old mice. The ratios of total adipose (*G*) and lean mass (*H*) over total body weight are shown as the mean ± standard deviation of 7 to 9 mice per group. *I*, daily food intake per 3-month-old mouse of indicated sex and genotype. Data derived from 1-week measurement are as mean ± standard deviation. n = 4.

To understand the physiological role that Hop2 plays in adipose tissue, we characterized mainly the visceral adipose phenotype in littermates of wt (wt or +/+), het (hets or +/−), and homozygous (−/−) mice. qRT–PCR and Western blot analyses of VAT at 2 months of age showed a 45% and 100% decrease in *Hop2* expression in *Hop2*^+/−^ and *Hop2*^−/−^ animals, respectively (Fig. 4, *A* and *B*), confirming the success of *Hop2* ablation and endogenous mRNA and protein expression of Hop2 in adipose tissues *in vivo*. Pups were born at Mendelian ratio with no overt developmental abnormalities in both the *Hop2*^+/−^ and *Hop2*^−/−^ mice up to 2 months of age. However, *Hop2*^−/−^ mice gained more weight than wt or het littermates when fed with a standard chow containing 14% fat by calories, which was progressively more severe with advancing age (Fig. 4*C*). Echo-MRI quantification revealed that *Hop2*^−/−^ mice display a 25% increase in total adipose tissue volume and a 30% increase in VAT volume compared with controls in both males and females at 6 months of age (Fig. 4, *D*–*H*). There is significant difference in size and weight of several vital organs such as the liver and kidney (Fig. S3, *A* and *B*). Het ablation of *Hop2* did not cause any fat abnormalities; we thus used both wt and het animals as controls for all the parameters examined in this study. H&E and ORO staining of the liver sections for neutral lipids that accumulate within the cells showed no change in hepatic lipid accumulation (Fig. S3*A*). Random and fasting glucose measurements of tail tip blood showed no difference between *Hop2*^−/−^ and control mice (Fig. S3*B*). Food consumption measurement and movement pattern monitoring showed no significant difference in the food intake between *Hop2*^−/−^ and control mice (Fig. 4*I*; data not shown). Collectively, these data demonstrate that global deletion of *Hop2* leads to increased adiposity, in addition to the previously reported infertility (34) and low bone mass defects in adult mice (28).

### Lack of Hop2 leads to an increased cell size of adipocytes

**Figure 5:**
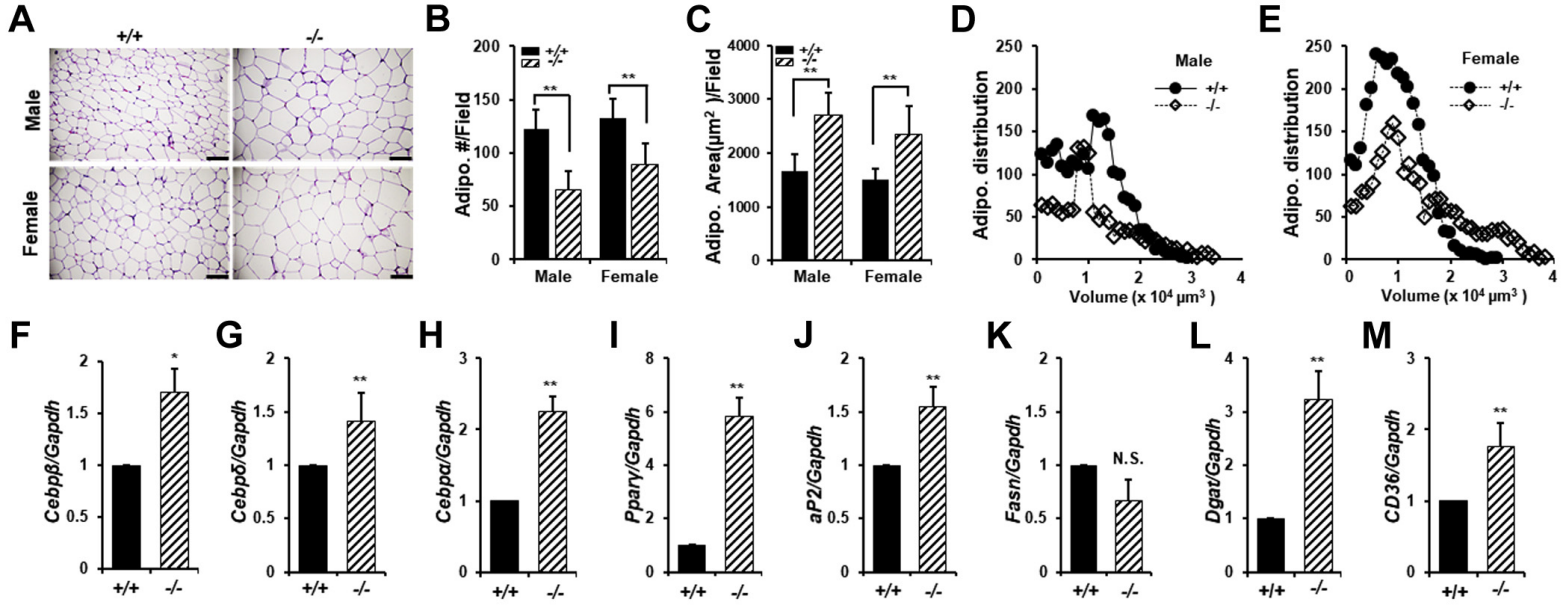
***Hop2* deficiency leads to increased size and volume of adipocytes.***A*, H&E staining of gonadal fat sections from 6-month-old mice of indicated sex and genotype. The *black bars* equal 100 μm. *B*, microscopic quantification of total cell number of adipocytes per field on H&E-stained adipose sections shown in *A*. *C*, microscopic quantification of adipocyte area per field on H&E-stained adipose sections shown in *A*. *D* and *E*, adipocytes' volume distribution of H&E-stained gonadal adipose sections is shown in *A* (5 μm in thickness) using ImageJ software. *F*–*M*, quantitative RT–PCR analysis of visceral adipose RNAs extracted from 6-month-old male mice for indicated adipocyte marker genes. Data are shown as mean ± standard deviation of three samples per genotype.

To discern whether the increase of adipose volume in *Hop2*^−/−^ mice is caused by increased proliferation (hyperplasia) and/or differentiation (hypertrophy) of preadipocytes, we measured the size and number of adipocytes by histology. Quantification of H&E-stained section of VAT revealed a 38% and 35% increase in the average adipocyte size, which corresponds to a 46% and 33% decrease in the number of adipocytes per field in *Hop2*^−/−^ mice of male and female, respectively, compared with controls (Fig. 5, *A*–*C*). Cell size distribution analysis found a greater number of *Hop2*^−/−^ adipocytes having a cell volume of 2.5 × 10^4^ to 3.0 × 10^4^ μm^3^, whereas most of the control adipocytes have a cell volume less than 10^4^ μm^3^ (Fig. 5, *D* and *E*), confirming that *Hop2*^−/−^ adipocytes are hypertrophic. No difference between *Hop2*^−/−^ and control VAT in the total DNA content, an indicator of total cell number, was observed (data not shown). Therefore, these results suggest that a generalized adipose volume increase in *Hop2*^−/−^ mice is primarily resulting from enhanced adipocyte differentiation and lipid accumulation. Consistently, qRT–PCR of total VAT RNA revealed an overall increase ranging twofold to fourfold in the level of late adipogenic marker genes, including *CEBPα*, *PPARγ*, *aP2*, diglyceride acyltransferase (*Dgat*), and cluster of differentiation 36 (*CD36*) known to be required for triglyceride storage and energy homeostasis, in *Hop2*^−/−^ mice compared with controls. A small increase ranging from 1.3- to 1.7-fold in the level of early adipogenic marker genes, such as *CEBPβ* and *CEBPδ*, is also present in mutant VAT samples (Fig. 5, *F*–*M*). In summary, histological and gene expression studies indicate that Hop2 is required for adipocyte phenotype acquisition.

### Loss of Hop2 leads to increased adipocyte differentiation cell autonomously

**Figure 6:**
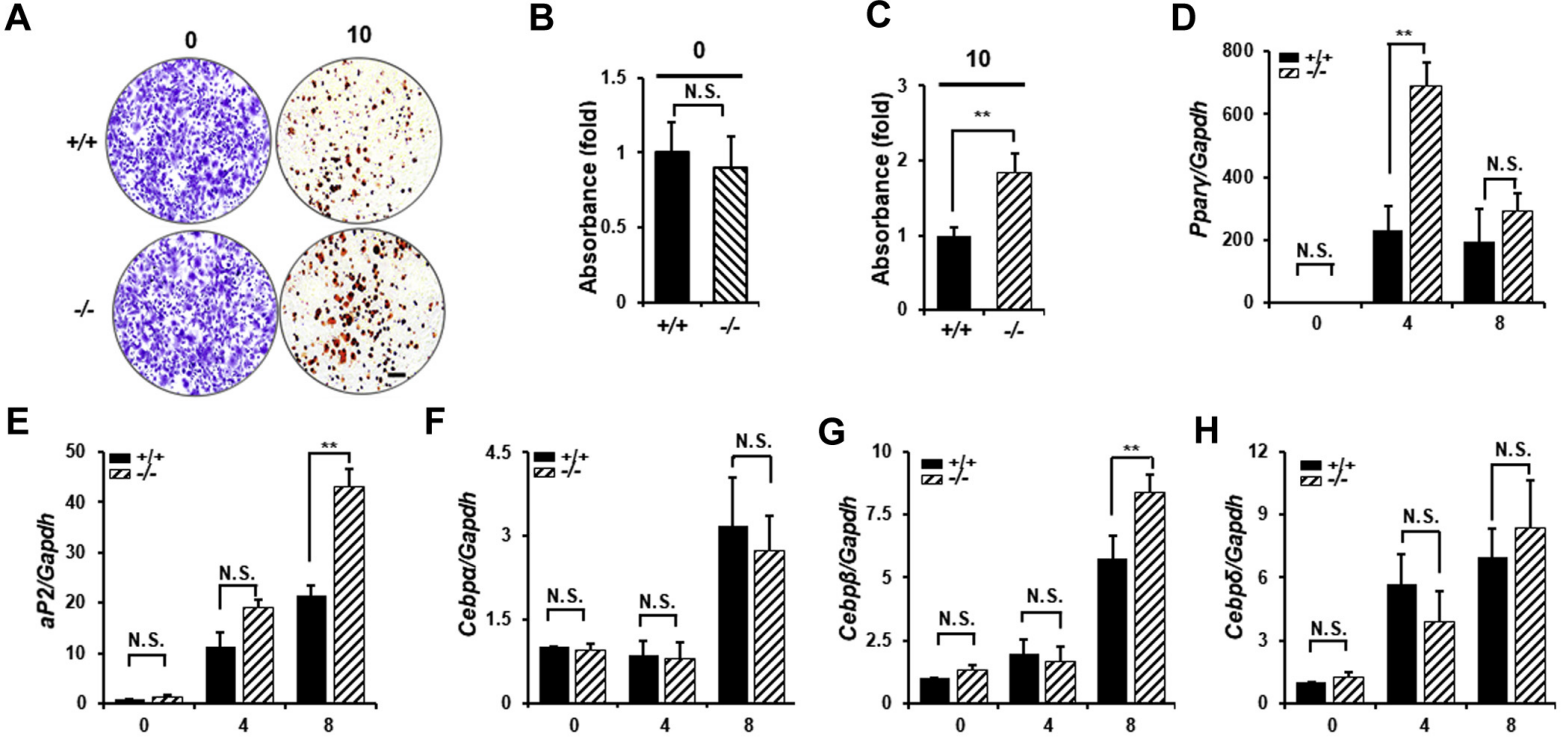
***Hop2***^**−/−**^**adipose-derived mesenchymal stem cells (aMSCs) display accelerated adipocyte differentiation.***A*–*C*, crystal violet staining (*A*, *left panels*) for equal seeding and Oil Red O (ORO) staining (*B*, *right panels*) for aMSC cultures following 10 days of adipogenic induction. Quantification of eluted dye from stained cells in *A*. Absorption of the eluate was measured photometrically at 570 nm for crystal violate (*B*) and at 510 nm for ORO (*C*). *Black bar* equals 200 μm. *D*–*H*, quantitative RT–PCR analysis of indicated adipocyte marker genes during differentiation of aMSCs for indicated days.

To exclude a potential systemic effect on lipid accumulation in *Hop2*^−/−^ adipocytes, we performed *ex vivo* adipocyte differentiation assays on isolated VAT-derived stromal cells (aMSCs). ORO staining of aMSC cultures at day 10 following adipogenic induction showed an 83% increase in the number of ORO+ colonies in *Hop2*^−/−^ aMSCs compared with controls (Fig. 6, *A*–*C*). As expected, there is an overall rise in adipocyte marker gene expression from day 0 to day 8 upon adipogenic induction. Consistent with a suppressive effect on differentiation, the loss of Hop2 in aMSCs leads to a greater increase in late adipocyte marker gene expression. Specifically, the level of *PPARγ* and *aP2* expression increased 230-fold in day 0 and 21-fold in day 8 control aMSC cultures, whereas 690-fold in day 0 and 43-fold in day 8 *Hop2*^−/−^ aMSC cultures, indicating that loss of Hop2 leads to 197% and 104% augmentation in the expression of these late adipocyte differentiation markers (Fig. 6, *D* and *E*). There is no significant difference between *Hop2*^−/−^ and control in the rise in the expression level of early adipocyte marker genes, such as *CEBPβ* and *CEBPδ*, in day 0 to day 8 aMSC cultures (Fig. 6, *F*–*H*). These data support a conclusion that loss of Hop2 predominantly impacts terminal adipocyte differentiation.

### Hop2 suppresses the DNA-binding activity of CEBPα

**Figure 7:**
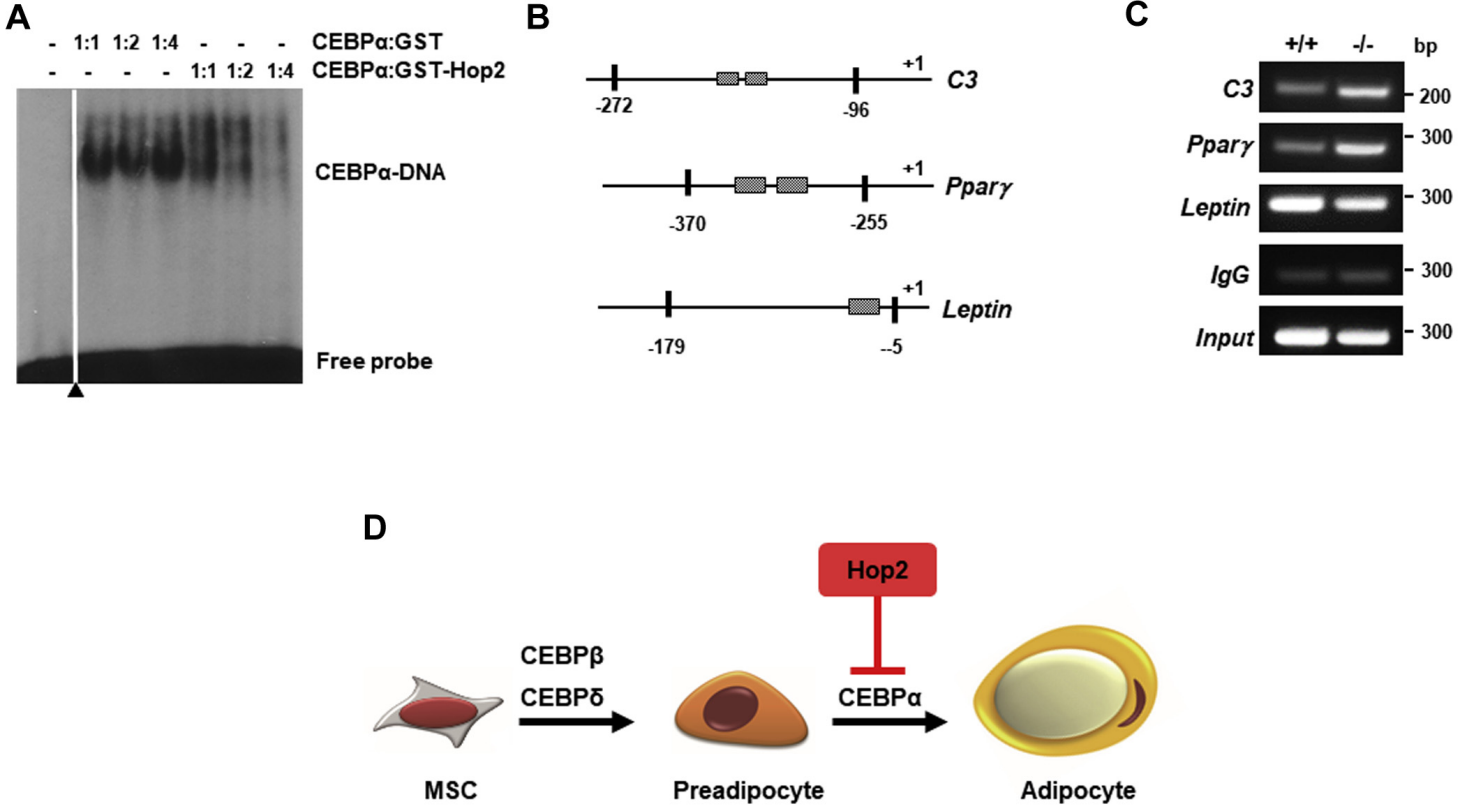
**Hop2 inhibits CEBPα to bind target DNA.***A*, EMSA for DNA-binding activity of CEBPα. Purified bacterially expressed His-CEBPα was incubated with increasing amount of purified GST-Hop2 or GST control and a synthetic palindrome of CEBP consensus DNA sequence as probe. *Black arrowhead* indicates one control lane removed from the original image for clarity. *B*, the location of primers used in chromatin immunoprecipitation (ChIP). *C*, ChIP assay of adipose tissues from wt and *Hop2*^−/−^ mice for promoter-binding activity of indicated CEBPα target genes. Primers used are listed in Table 1. One representative result of IgG negative control and input positive control of nonbinding sequence for all tested genes is shown. *D*, a working model. Hop2 dimerizes with CEBPα in adipocytes to suppress the CEBPα-mediated transactivation and adipocyte differentiation. CEBPα, CCAAT enhancer binding protein alpha; GST, glutathione-*S*-transferase.

Having established an essential function of Hop2 using both gain-of-function and loss-of-function models, we then wished to further determine the molecular mechanisms by which Hop2 suppressed adipocyte differentiation. To this end, we tested whether Hop2 inhibits the DNA-binding activity of CEBP proteins by EMSA and ChIP. A double-stranded oligo consisting of a synthetic CEBP consensus binding palindrome, tgcagATTGCGCAATctgca, was [^32^P]-labeled and used as a probe in EMSAs. As expected, purified His-CEBPα shifted the probe forming a protein–DNA complex (*solid arrowhead*) above the free probe (*empty arrow*) (Fig. 7*A*). However, the intensity of the His-CEBPα–DNA complex is reduced in the presence of an increasing amount of purified GST-Hop2 at the indicated ratio over a constant amount of His-CEBPα; the intensity of the protein–probe complex is reduced in a dose-dependent manner (Fig. 7*A*). However, the same amounts of purified GST do not affect the DNA-binding activity of His-CEBPα, indicating that Hop2 suppresses the DNA-binding activity of CEBPα *in vitro*. To confirm this is the case *in vivo*, we isolated NEs from VAT of 3-month wt and *Hop2*^−/−^ male mice for ChIP assays using Abs against CEBPα. PCR of CEBPα-bound genomic DNA revealed an increased binding activity of CEBPα to *C3* and *PPARγ* target genes in VAT from *Hop2*^−/−^ mice compared with wt controls (Fig. 7, *B* and *C*). From these data together with the rest of the findings presented in this study, we present a working model explaining that Hop2 negatively controls adipocyte differentiation by binding and suppressing the transactivation potential of CEBPα (Fig. 7*D*).

## Discussion

Terminal adipocyte differentiation is a process in which lineage-committed preadipocytes acquire characteristics such as the machinery for lipid transport and synthesis, insulin action, and secretion of adipocyte-specific proteins. As briefly reviewed in the introduction of this study, activation of CEBPβ and CEBPδ marks the initial stage of adipocyte differentiation, whereas expression of CEBPα and PPARγ induced by the heterodimer of CEBPβ:CEBPδ occurs in the mid and late stages of adipocyte differentiation. Each of the CEBPα and PPARγ can dimerize with other TFs to control the expression of a series of enzymes and proteins required for lipid synthesis, transport, and storage. In this study, we have taken a candidate approach and identified that Hop2 is an intrinsic factor suppressing adipocyte differentiation, likely though inhibits CEBPα to bind its target DNA.

Supporting a novel function of Hop2 in adipose, its mRNA is expressed in both brown and white adipose tissues at a higher level than all other tissues examined except for the testis, a tissue in Hop2 was originally discovered (44). Furthermore, the expression levels of Hop2 mRNA and protein are high in undifferentiated primary aMSCs and 3T3-L1 cells but progressively decrease to nearly undetectable in fully differentiated adipocytes. A similar change in the dynamic and temporal expression pattern has also been observed in other differentiation suppressors such as vimentin on osteoblasts (45). We thus speculate that a downregulation or removal of Hop2 is a prerequisite for the initiation of the adipocyte differentiation program. In agreement with a suppressive role, genetic deletion of *Hop2* in mice leads to accelerated adipocyte differentiation as demonstrated by a marked increase in the size of adipocytes, the volume of adipose tissue, and the level of adipocyte marker gene expression. Given the fact that the level of Hop2 expression in adipose tissues is much higher than that in other tissues, except for the testis, we believe that the activity of Hop2 in adipose is intrinsic. This conclusion is also supported by the observation that isolated aMSCs from *Hop2*^−/−^ mice display an accelerated adipocyte differentiation with a higher level of adipocyte marker gene expression and lipid accumulation compared with aMSCs from wt mice. Conversely, a gain of Hop2 function by overexpressing it in 3T3-L1 preadipocytes inhibited adipocyte differentiation, accompanied by reduced lipid accumulation and adipocyte marker gene expression. Thus, our *in vitro* and *in vivo* data presented in this study demonstrate a negative role that Hop2 plays in the regulation of adipocyte differentiation.

Molecularly, an ectopic expression of Hop2 suppresses CEBP-dependent transactivation of reporter genes on all three isoforms of adipocyte bZips, we infer that Hop2 is likely to function as a suppressor by binding and suppressing the activity of CEBPβ or CEBPδ isoforms in adipocytes. Supporting this speculation, an interaction is detected between endogenous Hop2 and CEBPβ in the NEs of VAT as well as between ectopically expressed Hop2 and CEBPδ in the NEs of COS1 cells. And it appears that the binding of Hop2 to the CEBPs prevents the latter to bind its target DNA, as demonstrated by EMSA in which Hop2 reduces the DNA-binding activity of CEBPα and by ChIP assays in which lacking Hop2 in adipocytes increases the binding of CEBPα to its target genes, such as *C3* and *Pparγ*. Whether the same Hop2 suppresses the CEBPβ- and CEBPδ-mediated transactivation *via* the same molecular mechanisms is currently unknown. Based on the observations, (1) a high level of Hop2 expression in undifferentiated aMSCs and 3T3-L1 cells; (2) sequential and transient expression patterns of CEBPα, CEBP-β, and CEBP-δ with the *CEBPβ/δ* being expressed a few hours following adipogenic induction, whereas *CEBPα* being expressed following the downregulation of *CEBPβ/δ* (10); and (3) a high homology (>90%) in the bZip motifs of all three adipocyte bZip isoforms, we thus propose that Hop2 may bind endogenous *CEBPβ/δ* at early stages of adipocyte differentiation while these two proteins are highly expressed. Studies are also necessary to further dissect whether Hop2 has different affinity and selectivity in its dimerization partner to each of the adipocyte bZip isoforms in adipocytes. It is also worth testing whether Hop2 could interact directly with PPARγ, a member of the NR superfamily, to affect adipocyte differentiation, although this possibility is low given that Hop2 coactivates, rather than suppresses, the transactivation potentials of several adipogenic NRs (44).

The present study expands a functional repertoire of Hop2 as a suppressor of the bZip TFs in adipocytes, in addition to previously known coactivators of other bZip TFs, such as ATF4 in osteoblasts (28) and NRs in to be identified hormone-secreting cells (44, 46). This context-dependent activity is likely attributed to functional domains outside the Zip motif of Hop2 as Zip structures have a well-defined role in mediating protein–protein interactions (Fig. S4). Indeed, it has been revealed by deletional mutagenesis that the Zip of Hop2 is responsible for binding to both the N- and C-Zip domains of ATF4 (28), Zip domain of CREB, as well as the DNA-binding domain of several NRs, including glucocorticoid receptor, estrogen receptor α and β, thyroid hormone receptor, androgen receptor, and progesterone receptor (44). Our demonstrations that Hop2:ATF4 dimerization stabilizes ATF4 in osteoblasts without affecting DNA binding of ATF4, whereas Hop2:CEBPα interaction inhibits the DNA binding of CEBPα without altering its protein expression, make it necessary to further dissect functional domains to understand fully a pathophysiological function of Hop2. It is worth noting that a cytoplasmic form of mutant HOP2 is highly expressed in many cell types of the mesenchymal lineage, including pericytes, adipocytes, fibroblasts, smooth muscle cells, and myoepithelial cells in human breast cancer samples (46), and relevant to the major finding of this study, the increased cytoplasmic HOP2 expression is associated with increased adipogenesis. These observations in human tissues thus further support a testable hypothesis that Hop2 is required intrinsically to suppress precocious adipocyte differentiation.

In summary, this study further expands our understanding of the function of Hop2, from a meiotic-specific factor to a TF necessary for the terminal differentiation or function of somatic tissues, which provides an example of how one gene can be involved in different cell lineages and cellular processes at different stages of life. Our findings broaden our understanding of the molecular mechanisms by which bZip TFs regulate diverse biological processes (21, 47, 48, 49). It is expected that our findings in this report will also elicit new research avenues for discovering novel mechanisms that control a reciprocal relationship between bone and fat in aging individuals (50, 51) and in patients with metabolic syndrome (52) or osteoporosis (53, 54).

## Experimental procedures

### Animal and cell culture

All animal experiments were performed following the Animals Scientific Procedures Act of 1986 and were approved by the local Ethical Review Committee of UTHealth. *Hop2*^−/−^ mice were generously provided by Dr Petukhova (the National Institutes of Health). All mice were housed in individually high-efficiency particulate air–filtered cages with sterile bedding, nesting, and free access to sterilized food and water. Strict mouse littermates were used in this study. All the cell lines were purchased from the American Type Culture Collection. Cell culture media were from Hyclone. COS1 monkey kidney cells and 3T3-L1 preadipocytes were cultured in Dulbecco's modified Eagle's medium supplemented with 10% fetal bovine serum (Atlanta Biologicals) and 1% penicillin–streptomycin (Gibco and Thermo Fisher Scientific). All cells were incubated at 37 °C and 5% CO_2_. 3T3-L1 cells permanently overexpressing Hop2 were selected with G418 (0.4 mg/ml). aMSCs were isolated from abdominal adipose that was minced followed by digestion with 0.1% collagenase type I (Worthington Biochemical Corporation) in PBS containing 1% antibiotics for 60 min at 37 °C. Collagenase was then neutralized followed by centrifugation for 5 min at 2000 rpm and additional wash with culture medium. The pellet containing aMSCs was suspended in a culture medium and cultured at 37 °C in 5% CO_2_.

### Pull-down, co-IP, colocalization, and Western blot analyses

Bacterially expressed His-tagged CEBPα, His-ATF4, and GST-Hop2 fusion protein were purified according to the method developed by Novagen and as described (28, 45, 55). Purified His-CEBPα (1 μg) was incubated with Ni–NTA-agarose (Qiagen) in PBS, pH 8.0, containing 10 mM imidazole at 4 °C with rotation for 1 h and then washed three times with PBS containing 15 mM imidazole. GST-Hop2 (1 μg) was then added and incubated with rotation for 2 h at 4 °C. After washing three times with PBS containing 50 mm imidazole, proteins were resolved in SDS-PAGE and stained with Coomassie brilliant blue before imaging. Conversely, purified GST-Hop2 bound on glutathione-sepharose 4B (GE17-0756-01; Sigma) was incubated with purified His-C/EBPα (1 μg) for 2 h in PBS, pH 7.4 with rotation at 4 °C. Following three times of washing with PBS, bound protein was resolved in SDS-PAGE.

For co-IP, COS1 cells with 85% confluence in 10 cm culture dishes were transfected with HA-Hop2, Flag-CEBPα, or both expression plasmids (5 μg each) using Lipofectamine (Invitrogen). NEs were prepared according to the methods described (36) and incubated with 5 μl of anti-Flag M2 beads (F3165; Sigma) or anti-HA (ab9110; Abcam) and 20 μl of IgA/G beads (Santa Cruz) in radioimmunoprecipitation buffer for overnight (ON) at 4 °C. After washing three times with radioimmunoprecipitation buffer, immunocomplexes and 10% of NEs as input controls were resolved by SDS-PAGE, transferred onto nitrocellulose membranes, and revealed by Western blotting using the corresponding Abs.

For Hop2 expression and colocalization of Hop2 and CEBPα, immunofluorescence was performed on frozen sections of freshly embedded adipose tissues and parafilm-embedded testis from 2-month-old wt and *Hop2*^−/−^ male mice. Immunohistochemistry was performed on deparaffinated sections (5 μm) of testis for Hop2 as a positive control. Following ON incubation with primary Abs, anti-Hop2 (1:100; Biorbyt; orb395150) and anti-CEBPα (1:100; Cell Signaling; 8178s), secondary Abs, antimouse IgG Alexa Fluor@488 (Abcam; ab150116), and goat anti-rabbit IgG Alexa Fluor@549 (Abcam; ab150079) were used to reveal positive signals. Images were captured with a laser scanning confocal microscope (LSM800; Carl Zeiss) following the manufacturer's instructions. For colocalization of HA-Hop2 and Flag-CEBPα, COS1 cells were transiently transfected with Flag-CEBPα and HA-Hop2 expression plasmids in eight chamber slides. Immunofluorescence was performed with anti-HA (1:100; Abcam; ab9110) and anti-Flag (1:100; Sigma; F3165) 48 h following transfections. Positive signals were revealed following detection with secondary Abs and imaged as described previously. Immunofluorescence of 3T3-L1 stably expressing HA-Hop2 for colocalization of Hop2 and endogenous CEBPα was also performed following a similar procedure described previously with anti-HA and anti-CEBPα on differentiating cultures at day 4 after adipogenic induction.

### DNA transfection, EMSA, and ChIP assay

COS1 cells were seeded at a density of 5 × 10^4^/well in 24-well plates and transfected with 0.2 μg of the reporter plasmid (*pC3-Luc* or *pLeptin-Luc*), 0.05 μg of β-galactosidase, 0.2 μg of TF plasmid (pCMV5-CEBPα, pCMV5-CEBP-β, or pCMV5-CEBP-δ) with or without 0.2 μg of pcDNA3.1-Hop2 using Lipofectamine (Invitrogen). Cells were lysed 24 h later, and the luciferase activity was normalized to the β-galactosidase activity. Each experiment was performed in triplicates and repeated at least three times. For EMSA, purified His-CEBPα and increasing amounts of GST-Hop2 or GST were incubated with 5 ρmol of a radiolabeled, self-annealed, and double-stranded oligonucleotide 5′-GATCTGCAGATTGCGCAATCTGCA-3′ as a probe at room temperature for 10 min. EMSA was performed as described (36, 55). ChIP assays of VAT from wt and *Hop2*^−/−^ mice were performed with a kit following instructions of the manufacturer (EMD Millipore; catalog no. 17-295). In brief, fresh adipose tissues were homogenized into small pieces, and lipid droplets floating at the top were removed following brief centrifugation. Homogenized tissues were crosslinked with 1% formaldehyde at room temperature for 15 min. Following washing, lysing, and sonicating, tissue lysate was precleared with protein A agarose/salmon sperm DNA for 30 min. Anti-CEBPα (Cell Signaling; 8178s) was then added to precleared cell lysate (2 mg/ml). Following ON incubation at 4 °C with rotation, protein A agarose/salmon sperm DNA was added to capture the immunocomplex for an additional 30 min. The precipitated DNA by anti-CEBPα was then eluted, and PCR was performed using primers listed in Table 1.

**Table 1:** Sequence of primers

Gene	GenBank no.	Primer sequence (5′-3′)
*CEBPβ*	NM_009883	AAGCTGAGCGACGAGTACAAGA
		GTCAGCTCCAGCACCTTGTG
*CEBPδ*	NM_007679	TCCACGACTCCTGCCATGTA
		GCGGCCATGGAGTCAATG
*CEBPα*	NM_007678	AGGTGCTGGAGTTGACCAGT
		CAGCCTAGAGATCCAGCGAC
*PPARγ*	U09138	TCGCTGATGCACTGCCTATG
		GAGAGGTCCACAGAGCTGATT
*AP2*	NM_024406	AAGGTGAAGAGCATCATAACCCT
		TCACGCCTTTCATAACACATTCC
*FASN*	NM_007988.3	ACC TCT CCC AGG TGT GTG AC
		ACC TCT CCC AGG TGT GTG AC
*DGAT1*	NM_010046.3	GTA GAA GAG GAC GAG GTG CGA GAC
		GGG CTT CAT GGA GTT CTG GAT AGT
*CD36*	NM_001159558.1	TGG CCT TAC TTG GGA TTG G
		CCA GTG TAT ATG TAG GCT CAT CCA
PPARγ promoter		TTC AGA TGT GTG ATT AGG AG
		AGA CTT GGT ACA TTA CAA GG
Leptin promoter		TCG AGG ATT ACC GGC TCA TA
		GAA AGA CTG GTG GAG GAG AAA G
C3 promoter		ACC TGC CAC CTC CTA GA
		AGA GAT ATA CCT GAG TGT TGG AAT

### Body adipose composition, histology, and metabolic phenotype analysis

The total adipose tissue composition was measured by MRI, using an EchoMRI-100 QMR instrument (EchoMRI), at 6 months old before sacrifice. Gonadal adipose fat pads were dissected as described (56), from the mice of different genotypes, weighed, and immediately placed in cassettes, and submerged in 10% neutral buffered formalin. After being fixed ON, samples were dehydrated and embedded in paraffin. Tissues were sectioned (5 μm) with Leica RM216 Microtome and stained with H&E and imaged under Olympus BX51 fluorescent microscope with 20× magnification. Three representative portions of each slide were analyzed using Fiji imaging software with the Adiposoft,(version 1.13 plugin, downloaded from ImageJ software, https://imagej.net/plugins/adiposoft). Freshly isolated internal organs from 6-month-old wt and *Hop2*^−/−^ male mice were dissected, weighed, and recorded. Livers were collected and snap frozen in liquid nitrogen and embedded with OCT medium (Tissue-Tek), and frozen livers were sectioned at 5 μm on a Leica CM3050 cryostat set to −20 °C and then air dried. Rehydrated liver sections were placed in 100% propylene glycol for 2 min and stained with H&E and 0.5% ORO (Sigma–Aldrich).

### Gene expression analysis

For qRT–PCR, total RNA from different mouse adipose tissues or cells was isolated using TRIzol (Invitrogen) according to the manufacturer's protocols. cDNA was prepared using 0.5 μg total RNA, which were then diluted 10-fold for real-time PCR using SYBR green system (Invitrogen). Triplicates for each sample were performed in three independent experiments. mRNA levels in each sample were calculated for the mean Ct values, and data were normalized relative to the expression of GAPDH or β-actin. Levels of mRNA were expressed as fold induction using the 2^−ΔΔCt^ method. The primers used for qRT–PCR are shown in Table 1.

### Adipocyte differentiation assay

Confluent primary aMSCs at passage 1 or 3T3-L1 preadipocytes in 12-well plates were cultured for additional 2 days (defined as day 0) and adipogenic cocktail containing insulin (10 μg/ml), dexamethasone (1 μM), and 3-isobutyl-1-methylxanthine (IBMX; 0.5 mM) was added. On day 2, the medium was replaced with 10 μg/ml insulin only and replenished every 2 to 3 days until the end of the experiment. Crystal violet staining was performed to confirm the equal number of cells seeded on day 0. Lipid accumulation was assayed by ORO staining on day 4 and day 8 cultures and quantified by counting the ORO-positive colony numbers or spectrophotometry measuring absorbance at 570 nm.

### Statistical analysis

Statistical analyses on groups of two were performed using one-way ANOVA or the paired *t* test for the littermate comparison. A *p* value of <0.05 was considered to be statistically significant; ∗*p* < 0.05 and ∗∗*p* < 0.01 for all panels.

## Data availability

The data supporting this study are available in the article and from the corresponding author (Xiangli.y.elefteriou@uth.tmc.edu) upon request.

## Supporting information

This article contains supporting information.

## Conflict of interest

The authors declare that they have no conflicts of interest with the contents of this article.
