# Supplementary material for: Hop2 interacts with the transcription factor CEBPα and suppresses adipocyte differentiation
Source: J Biol Chem. 2021 Sep 30;297(5):101264. doi: 10.1016/j.jbc.2021.101264 (PMC8528721; doi:10.1016/j.jbc.2021.101264)

Legends for supplemental figures

**Supplemental Figure 1. Validation of Hop2 antibody.**

**A.** Immunofluorescence of frozen sections of adipose tissues using anti-Hop2 antibody.

**B.** Immunohistochemistry of testis sections from animals of indicated genotype using anti-Hop2 antibody.

**Supplemental Figure 2. Colocalization of overexpressed Hop2 and CEBPα.**

**A.** Immunofluorescence of COS1 cells transiently overexpressing Hop2 and CEBPα using indicated antibodies or Hoechst for nuclei.

**B.** Immunofluorescence of 3T3-L1 cells stably expressing HA-Hop2. Cells were induced to differentiate with adipogenic cocktail for 4 days. Antibodies used were anti-HA for Hop2 and anti-CEBPα for endogenous CEBPα. Scale bar = 100 μm for all panels except for the one in the right top and bottom panels in both **A** and **B**, which is 20 μm.

**C.** Western blot analysis of CEBPβ and CEBPδ in nuclear extracts from differentiating 3T3-L1 adipocytes.

**D.** Western blot analysis of CEBPα in nuclear extracts from differentiating 3T3-L1 adipocytes.

**E-F.** Co-IP and Western blot analysis of NEs from differentiated 3T3L1 cell for endogenous interaction of Hop2 with CEBPs . Antibodies used for IP and IB are indicated.

**Supplemental Figure 3. Metabolic phenotyping of 6-month-old** **mice.**

**A.** Image of indicated for the size of the organs from wt, *Hop2*+/-, and *Hop2*-/- male mice.

**B.** Quantification of organ weight indicated in **A**. n = 4.

**C**. Histology of paraffin (for H&E staining, top panels) and frozen liver sections (for Oil red O staining, ORO, bottom panels) of male mice of indicated genotypes.

**D.** Serum glucose level measurements of wt and *Hop2*-/- male mice**.**

**Supplemental Figure 4.** A model of Hop2 dimerizes with CEBPα and ATF4 (A). Working model of Hop2 during osteoblast and adipocyte differentiation(B).


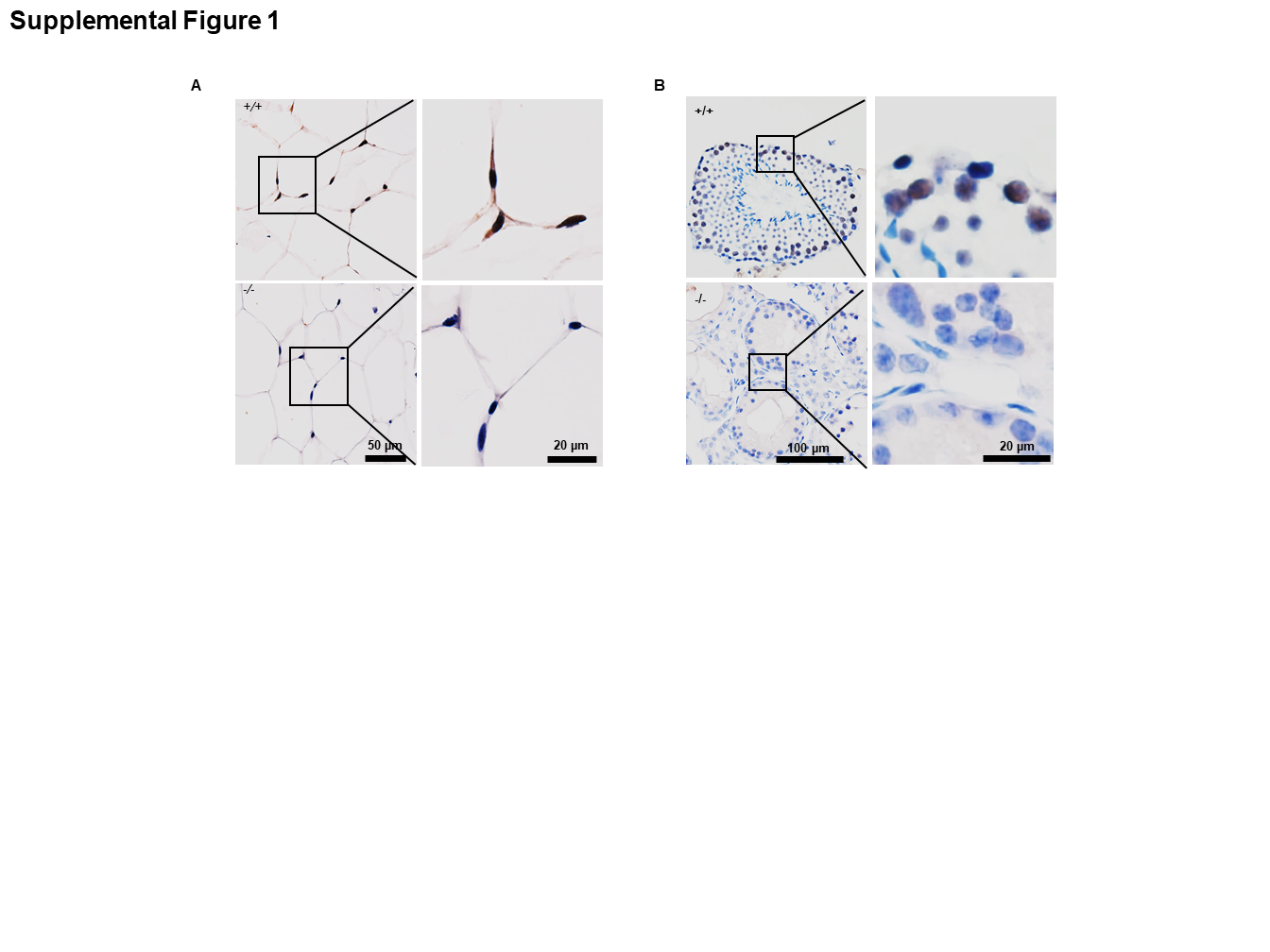


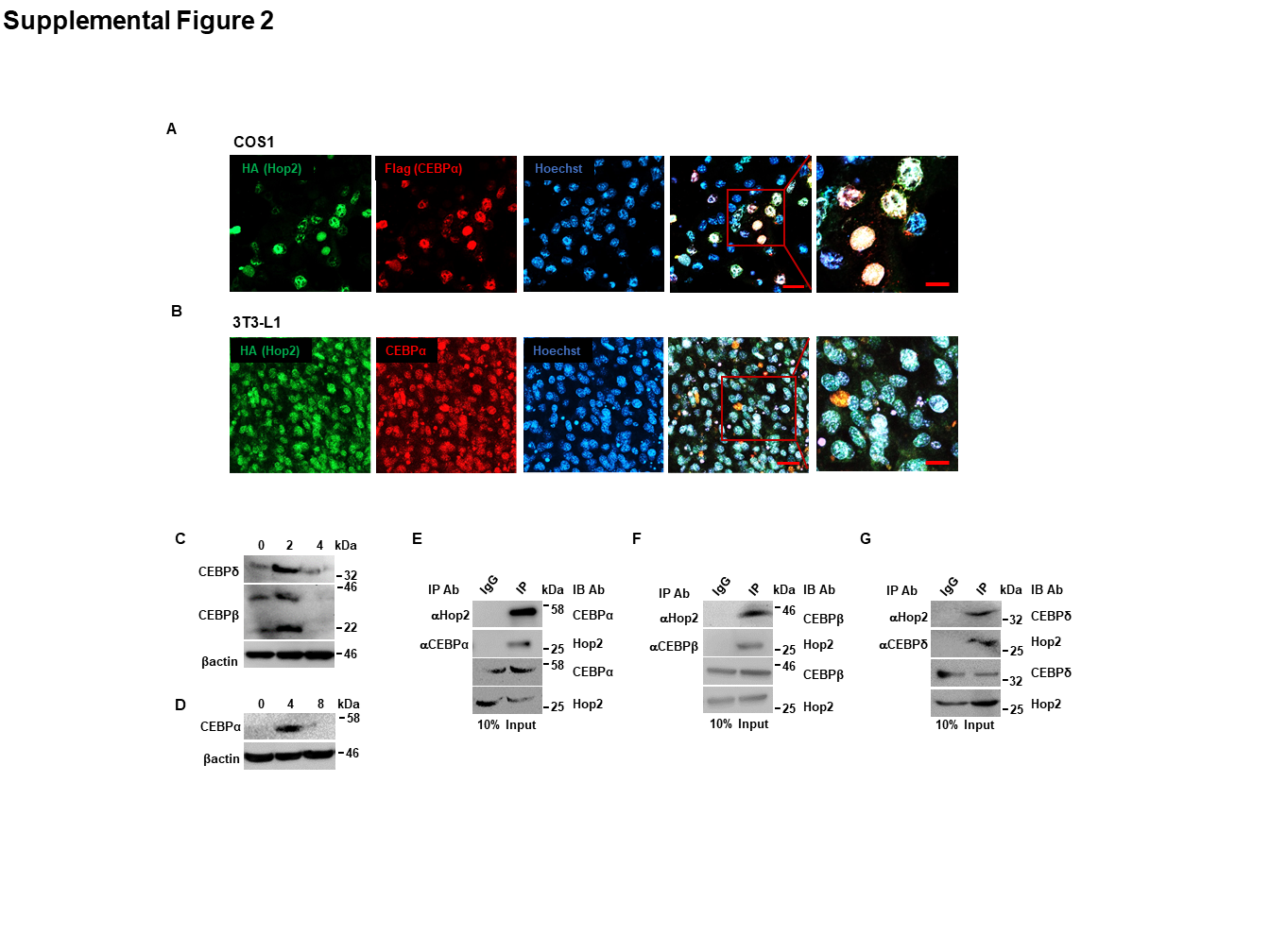


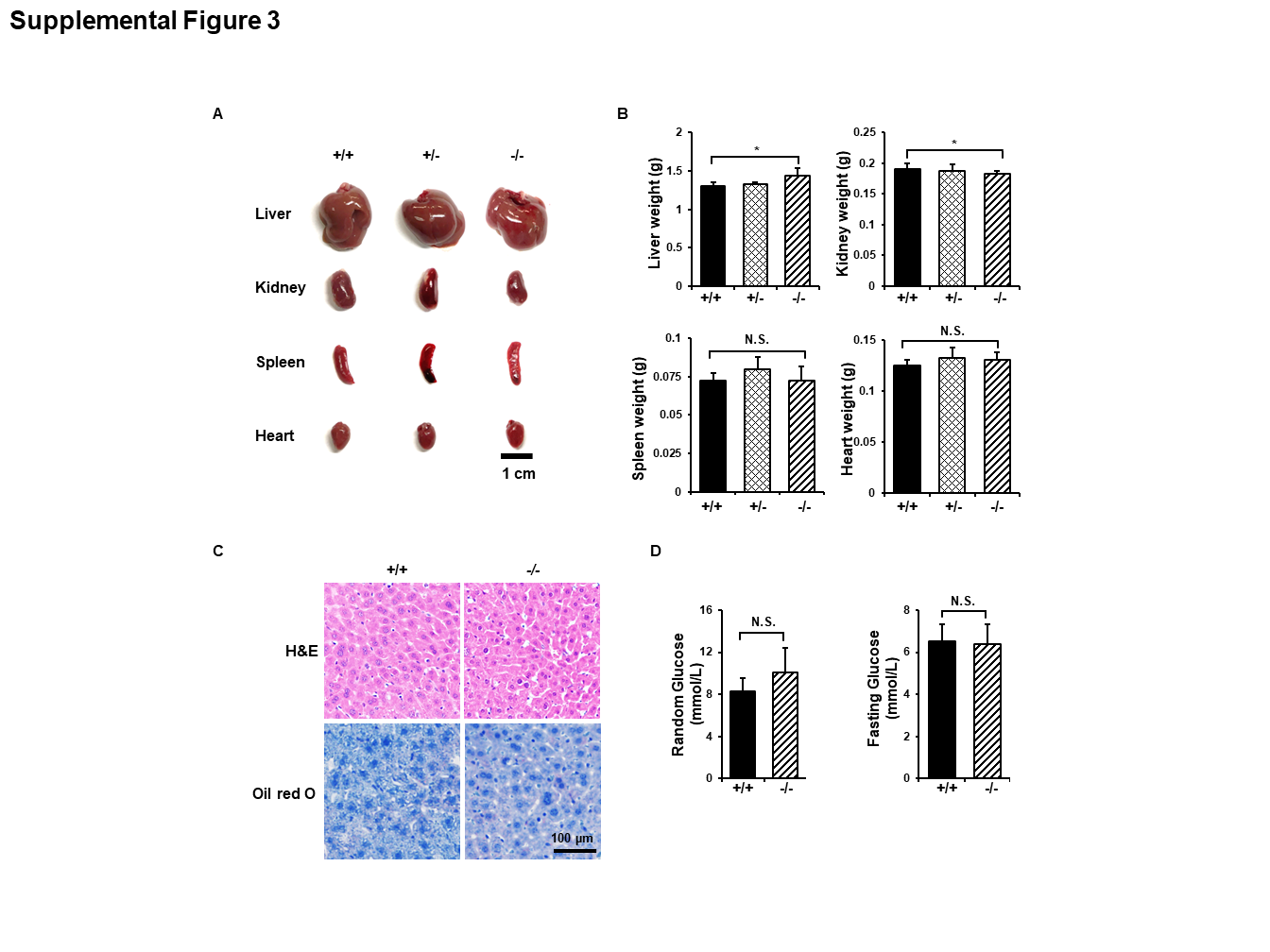


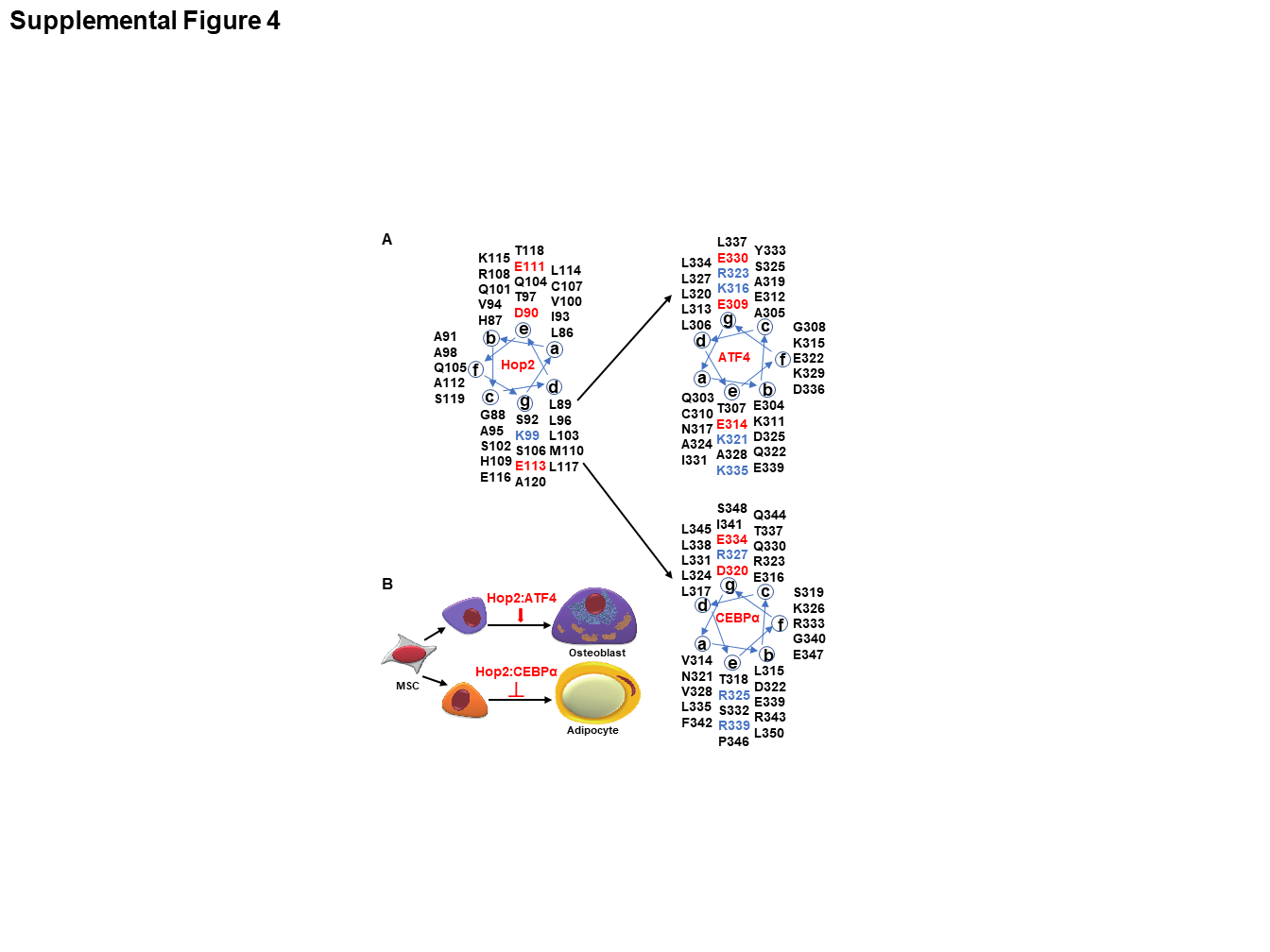

Supplement: Supplemental Figures S1–S4 [file mmc1.docx]
